# Spontaneous bending of pre-stretched bilayers

**DOI:** 10.1007/s11012-017-0732-z

**Published:** 2017-08-17

**Authors:** Antonio DeSimone

**Affiliations:** SISSA, via Bonomea 265, 34136 Trieste, Italy

**Keywords:** Pre-stretched bilayers, Thin plates and shells, Shape programming, Spontaneous curvature

## Abstract

We discuss spontaneously bent configurations of pre-stretched bilayer sheets that can be obtained by tuning the pre-stretches in the two layers. The two-dimensional nonlinear plate model we use for this purpose is an adaptation of the one recently obtained for thin sheets of nematic elastomers, by means of a rigorous dimensional reduction argument based on the theory of Gamma-convergence (Agostiniani and DeSimone in Meccanica. doi:10.1007/s11012-017-0630-4, 2017, Math Mech Solids. doi:10.1177/1081286517699991, arXiv:1509.07003, 2017). We argue that pre-stretched bilayer sheets provide us with an interesting model system to study shape programming and morphing of surfaces in other, more complex systems, where spontaneous deformations are induced by swelling due to the absorption of a liquid, phase transformations, thermal or electro-magnetic stimuli. These include bio-mimetic structures inspired by biological systems from both the plant and the animal kingdoms.

## Introduction

We discuss a two-dimensional (nonlinear) plate model to describe the spontaneously bent configurations of pre-stretched bilayer sheets that can be obtained by tuning the pre-stretches in the two layers. Our motivation is to try to acquire a thorough understanding of a model system (“easily” realised in a laboratory, and “easily” reproducible in numerical simulations based on the finite element method) to study shape programming and morphing of surfaces in a relatively simple and controllable setting. We plan to use the results obtained with the model system to address similar questions arising in more complex physical systems where heterogeneous spontaneous deformations are induced by swelling due to the absorption of a liquid (hydrogels [22–24, 29] and hygromoprphic structures [26]), or by phase transformations driven by applied thermal or electro-magnetic fields (shape-memory alloys [6]; liquid crystal elastomers [11, 13, 27, 31, 32]).

The study of shape changes induced by heterogeneous response to external stimuli in thin structures has a long history. Among the classical contributions regarding the thermal buckling of a bi-metal strip or disk, we cite [30] and [12]. A variant of this problem is the study of thermally-induced delamination and blistering of thin film coatings deposited over solid substrates, see, e.g., [14, 15] and the many references cited therein. While often the shape changes are detrimental to the correct functioning of a structure, such as in the blistering of coating films, they can be exploited to perform a function, like in the use of bimetallic strips as thermostats. And in recent years, the problem of exploiting material heterogeneities to induce controlled shape changes in response to an external, spatially homogeneous stimulus has received considerable attention.

Often, interesting solutions to this problem of shape programming are inspired by biological structures. In the realm of plants or animals, shape control is usually accomplished by growth (in which case shape evolution is slow) or by active stresses or distortions (a mechanism akin to muscle contraction, in which case shape evolution can be fast and used, for example, for locomotion or to trap prey insects in carnivorous plants). Morphogenesis, shape control and programming, and morphing of surfaces are by now classical research topics in Mechanics. The literature is vast, and growing at a very fast pace.

Inspired by [8, 28], we propose here the use of composite, layered thin films, made by pre-stretching and gluing latex sheets in various ways, to study the emergent shapes in a thin structure that relaxes to equilibrium by developing differential strains along the thickness. Also the case of spontaneous curvature arising form lateral (i.e, within the mid-plane) strain variations is interesting and important. This mechanism is based on the Theorema Egregium of Gauss, see, e.g. [5, 20], and this will be considered elsewhere since we focus here on homogenous pre-strecthing of each of the two halves of a symmetric bilayer. We show that by “engineering” the pre-stretch in each of the two layers, one can reproduce different structural models, such as the bistable shell and the shell with zero stiffness with respect to twisting discussed in [18] and [17], respectively. Moreover, by uniaxially pre-stretching one of the layers, and shaping the mid-plane of the bilayer in the form of a long and narrow rectangle, one can reproduce the system considerered in [8] by varying the angle between the long axis of the rectangle and the pre-stretch direction. We analyse the resulting tuneable helical ribbons, and the emergence of spontaneously curved shapes, with the help of a different model than the one considered in [8].

## Three-dimensional model for pre-stretched bilayers

Before proceeding, let us establish some general notation which will be used throughout. For the standard basis of $\mathbb {R}^3$ we use the notation $\{\mathsf {e}_1,\mathsf {e}_2,\mathsf {e}_3\}$. We denote by $\mathrm{M}^+(3)$ the set of $3{\times 3}$ matrices with positive determinant, $\mathrm{SO}(3)$ and $\mathrm{Sym}(3)$ are the sets of $3{\times 3}$ rotations and symmetric matrices, respectively. Similarly, for $2{\times 2}$ matrices, we use the symbols $\mathrm{SO}(2)$ and $\mathrm{Sym}(2)$ for rotations and symmetric ones. Moreover, $\mathrm{I}\in \mathrm{SO}(3)$ and $\mathrm{I}_2\in \mathrm{SO}(2)$ are the identity matrices in three and two dimensions, respectively. We write $\mathbb {I}_2$ for the identity map from $\mathrm{Sym}(2)$ to $\mathrm{Sym}(2)$ (a tensor of rank four), and use the symbol $\mathrm{tr}^2A$ for the square of the trace of a matrix *A*.

The reference configuration of our (symmetric) bilayer film is denoted by $\Omega _h:=\omega \times (-h/2,h/2)\subset \mathbb {R}^3$, where $\omega \subset \mathbb {R}^2$ is the mid-surface and *h* is the (total) thickness. Each of the two halves of the bilayer has thickness *h* / 2. For a point $x\in \Omega _h$ we write $x=(x_1,x_2,x_3)$ and $x=(x',x_3)$, with $x'=(x_1,x_2)\in \omega$ and $x_3\in (-h/2,h/2)$. We are interested in deformations $y:\Omega _h\rightarrow \mathbb {R}^3$ minimising the 3d elastic energy of the system

2.1 $$\begin{aligned} y_h=\mathop {{{\mathrm{arg\,min}}}}\limits _{y\in {\mathscr {A}}} \int _{\Omega _h} W_h(x_3, \nabla _x y(x))\mathrm{d}x \end{aligned}$$

where ${\mathscr {A}}$ is a suitable function space, $\nabla _x y(x)$ is the deformation gradient at *x*, and

2.2 $$\begin{aligned} W_h(x_3, F)= W_0(FU^*_h(x_3))\,. \end{aligned}$$

Here $W_0\ge 0$ is a frame-indifferent isotropic energy density such that $W_0(G)=0$ implies that $G\in \mathrm{SO}(3)$. Moreover,

2.3 $$\begin{aligned} U^*_h(x_3)= \mathrm{I}+hH^*(x_3) + o(h) \end{aligned}$$

with *o*(*h*) an “error” term going to zero faster than *h* as $h\rightarrow 0$, and

2.4 $$\begin{aligned} H^*(x_3)= \left\{ {\begin{array}{ll} {H^*_1 }&\quad {\hbox { if } x_3\in [0,h/2)}\\ {H^*_2} &\quad {\hbox { if }x_3\in (-h/2,0)} \end{array} }\right. \end{aligned}$$

where $H^*_1$, $H^*_2$ are constant $3{\times 3}$ symmetric matrices.

The matrix $U^*_h$ has the physical meaning of an $x_3$-dependent pre-stretch. Indeed,

2.5 $$\begin{aligned} \overline{F}=\mathop {{{\mathrm{arg\,min}}}}\limits _{F\in \mathrm{M}^+(3)} W_0(FU^*_h)\Rightarrow \overline{F}=R \overline{U}\,,\quad R\in \mathrm{SO}(3) \end{aligned}$$

where

2.6 $$\begin{aligned} \overline{U}=\overline{U}_h(x_3)=(U^*_h(x_3))^{-1}= \mathrm{I}-hH^*(x_3) + o(h) \end{aligned}$$

meaning that the state of deformation that minimises the energy density is the inverse of (the pre-stretch) $U^*_h$, together with all the states obtained from this one by superposing a rigid body rotation. This deformation brings back the material to the unstretched, natural (stress-free) configuration of minimal (zero) energy density. In assuming that the thickness of the two layers composing $\Omega _h$ is *h* / 2, independent of the pre-stretch, we are ignoring the transversal contraction of the film occurring when the pre-stretch is applied, a small error at least for moderate pre-stretches^1^. By analogy with the literature on phase transforming solids, we will call $\overline{U}$ the preferred, or stress-free stretch (also called Bain stretch/strain in the case of martensitic transformations in crystalline solids, or spontaneous/preferred stretch/distortion; thermal dilatation/distortions would also be an example). Notice that, from (2.3) and (2.4), the pre-strain is $E^*_h= U^*_h - \mathrm{I} \, + o(h) = hH^*$, so that

2.7 $$\begin{aligned} E_h^*(x_3)= \left\{ \begin{array}{ll} E^*_1= h H^*_1 &\quad \hbox { if }x_3\in [0,h/2)\\ E^*_2= h H^*_2 &\quad \hbox { if }x_3\in (-h/2,0) \end{array} \right. \end{aligned}$$

A prototypical example for $W_0$ is a compressible version of the neo-Hookean energy density, namely,

2.8 $$\begin{aligned} W_0(F) \,:=\, \frac{\mu }{2} \Big [ |F|^2-3-2\log (\det F\,) \Big ] + W_{vol}(\det F\,) \ \end{aligned}$$

where $\mu >0$ is a material constant (elastic shear modulus) and the function $W_{vol}:(0,\infty )\rightarrow [0,\infty )$ is $\mathrm{C}^2$ around 1 and fulfills the conditions:

$$\begin{aligned} W_{vol}(t)=0 \iff t=1, \qquad W_{vol}(t)\longrightarrow \infty \ \,\mathrm{as}\ \,t\rightarrow 0^+, \quad W_{vol}''(1) >0. \end{aligned}$$

For example, $W_{vol}$ could be taken as $t\mapsto c\,(t^2-1-2\log t)$. Expression (2.8) is adequate for the (small strain) behaviour of latex sheets, and it is the one we will use for our developments. Setting

2.9 $$\begin{aligned} \gamma := \frac{W_{vol}''(1)}{2\mu +W_{vol}''(1)} \end{aligned}$$

we can represent the tensor of elastic constants (the second differential of $W_0$ evaluated at $F=\mathrm{I}$) in terms of the more familiar Young modulus *Y* and Poisson ratio $\nu$ using the relations

2.10 $$\begin{aligned} 2\mu =\frac{Y}{1+\nu }\,,\quad 2\gamma \mu = \frac{\nu Y}{1-\nu ^2}\,. \end{aligned}$$

## Small thickness limit and two-dimensional bending model

The model presented in the previous section falls within the framework analysed in [2, 3], where thin sheets of nematic elastomers are considered. These are soft phase transforming materials which, as a consequence of the isotropic-to-nematic phase transformation, develop preferred stretches of the form (2.6). They are described by elastic energy densities of the form (2.8). It is proved in [2, 3] that, in the thin limit $h\ll 1$, minimisers $y_h$ of the 3d energy (2.1) approach a function *y* with the following properties:

i. *y* is independent of $x_3$, so we write $y:\omega \rightarrow \mathbb {R}^3$;
ii. *y* is an isometric embedding of $\omega$ into $\mathbb {R}^3$ (an isometry of $\omega$), i.e., $\nabla 'y(x')^T \nabla 'y(x')=\mathrm{I}_2$ where $\nabla '$ is the gradient with respect to $x'$, so we write $y\in {\mathscr {A}}_\mathrm{iso}$, and ${\mathscr {A}}_\mathrm{iso}$ is a suitable function space of isometries of $\omega$;
iii. *y* minimises among isometries the 2d (bending energy) functional
  3.1 $$\begin{aligned} D\int _{\omega }\frac{1}{2} \Big [ (1-\nu )|A_y(x')-\overline{A}|^2 + \nu \mathrm{tr}^2(A_y(x')-\overline{A}) \Big ] \mathrm{d}x' \end{aligned}$$

where

$$\begin{aligned} D=\frac{Yh^3}{12(1-\nu ^2)} \end{aligned}$$

is the bending modulus,

$$\begin{aligned} A_y(x')=(\nabla 'y(x') )^T\nabla ' {\mathsf n} (x') \end{aligned}$$

is the curvature tensor, or second fundamental form of the surface $y(\omega )$, a $2{\times 2}$ symmetric matrix (here ${\mathsf n} (x')= \partial _{x_1} y (x') \wedge \partial _{x_2} y (x')$ is the normal to $y(\omega )$ at the point $y(x')$), and the *target curvature* $\overline{A}$ is given by

3.2 $$\begin{aligned} \overline{A}=\frac{3}{2}(\check{H}^*_2- \check{H}^*_1)= \frac{3}{2h}(\check{E}^*_2- \check{E}^*_1)\,, \end{aligned}$$

where $\check{E} \in \mathrm{Sym}(2)$ is the $2{\times }2$ symmetric matrix obtained from $E\in \mathrm{Sym}(3)$ by deleting its third row and third column.

Looking for configurations of minimal bending energy, in the absence of external loads and restricting attention to the case in which the target curvature $\overline{A}$ is constant, we look for deformations $y\in {\mathscr {A}}_\mathrm{iso}$ whose second fundamental forms are constant pointwise minimisers of the integrand of (3.1). This leads us to seeking surfaces with constant curvature $A_y\equiv A$, where $A\in \mathrm{Sym}(2)$ is a minimiser of the integrand of

3.3 $$\begin{aligned} D\int _{\omega }\frac{1}{2} \Big [ (1-\nu )|A(x')-\overline{A}|^2 + \nu \mathrm{tr}^2(A(x')-\overline{A}) \Big ] \mathrm{d}x' \end{aligned}$$

(3.1) satisfying the constraint $\mathrm{det} (A)=0$. In fact, it is well known that the isometry constraint implies that the Gaussian curvature *K* of $y(\omega )$ must vanish, i.e., $K=\mathrm{det} (A_y)=0$.

More in detail, our strategy to find configuration of minimal bending energy is to regard (3.1), which is a functional defined over isometric embeddings *y* of the flat domain $\omega$, as a functional (3.3) defined over symmetric-valued matrix fields $A(x')$ defined on $\omega$, satisfying the compatibility equations $\mathrm{det} (A)=0$ and $\partial _{\beta }A_{\alpha \sigma }-\partial _{\sigma }A_{\alpha \beta }=0$ (Gauss–Codazzi–Mainardi equations, trivially satisfied by a constant matrix field with zero determinant). These compatibility equations guarantee that the symmetric matrix field *A* is, in fact, the second fundamental form of an isometry *y*, i.e., $A(x')=A_y(x')$ for some isometry *y*: this is the content of the fundamental theorem of (the differential geometry of) surfaces, see [9]. The equivalence of the two minimisation problems, namely, the one of finding *y* minimising (3.1) or finding *A* minimising (3.3) is thus established.

Since, for $$A\in \mathrm{Sym}(2)$$,

$$\begin{aligned} \mathrm{det} A = \frac{1}{2} \Big [\mathrm{tr}^2 A - |A|^2\Big ] \end{aligned}$$

we can write the constraint $\mathrm{det} (A)=0$ with the help of a quadratic form

3.4 $$\begin{aligned} \mathrm{det} A =\frac{1}{2} \mathbb {D}A \cdot A =0\,, \quad \mathbb {D}=\mathrm{I}_2 \otimes \mathrm{I}_2 - \mathbb {I}_2 \end{aligned}$$

where $(\mathrm{I}_2 \otimes \mathrm{I}_2 ) A = (A\cdot \mathrm{I}_2 ) \mathrm{I}_2 = (\mathrm{tr}A) \mathrm{I}_2$. With a similar notation, we can write the integrand of (3.3) as

3.5 $$\begin{aligned} \frac{1}{2} \mathbb {C}(A-\overline{A})\cdot (A-\overline{A})\,, \quad\mathbb {C}= (1-\nu ) \mathbb {I}_2 + \nu (\mathrm{I}_2 \otimes \mathrm{I}_2)\,. \end{aligned}$$

We are thus led to the (finite-dimensional) constrained minimisation problem

3.6 $$\begin{aligned} \min _{A\in \mathrm{Sym}(2)} \frac{1}{2} \mathbb {C}(A-\overline{A})\cdot (A-\overline{A})\,, \quad \hbox {subject to: }\frac{1}{2} \mathbb {D}A \cdot A =0\,. \end{aligned}$$

Introducing a (scalar) Lagrange multiplier $\lambda$ associated with the isometry constraint (3.4), we have that critical points for problem (3.6) satisfy the equilibrium equations

3.7 $$\begin{aligned}\left\{ \begin{array}{l} M(A) := \mathbb {C}(A-\overline{A}) = \lambda \mathbb {D}A \\ \frac{1}{2} \mathbb {D}A\cdot A =0 \end{array} \right. \end{aligned}$$

where $M(A)\in \mathrm{Sym}(2)$ is the tensor of bending and twisting moments corresponding to the curvature tensor *A*.

## Spontaneously bent bilayers

Depending on the pre-stretch $U^*_h(x_3)$ in (2.3), or the pre-strain $E_h^*(x_3)$ in (2.7), different target curvatures $\overline{A}$ will result from formula (3.2). When $\mathrm{det} \overline{A}=0$, the obvious solution of the problem of minimal bending energy (3.6) is $A=\overline{A}$, and the target curvature $\overline{A}$ will be realised in practice. Thus, $\overline{A}$ will be the *spontaneous curvature* exhibited by the bilayer in the absence of external loads.

When $\mathrm{det} \overline{A}\ne 0$, the spontaneous curvature, i.e., the curvature tensor minimising the bending energy (3.1) (hence solving the minimisation problem (3.6)), will be different from $\overline{A}$. We discuss in what follows the prototypical cases obtained by solving the equilibrium equations (3.7), and retaining only the solutions of minimal bending energy density (stable equilibria). We omit the details since the calculations are straightforward. The same problem is solved in [19] with the help of a graphical construction.

### Uniaxial pre-stretch in the bottom layer

This is the case $\check{E}^*_1=0$, $\check{E}^*_2= \varepsilon ({\mathsf {e}_1}\otimes {\mathsf {e}_1})$, $\varepsilon >0$. We get from (3.2)

4.1 $$\begin{aligned} \overline{A}= \frac{3}{2h} (\check{E}^*_2 - \check{E}^*_1) = \overline{c} ({\mathsf {e}_1}\otimes {\mathsf {e}_1})\,, \quad \overline{c}= \frac{3 \varepsilon }{2 h} \end{aligned}$$

and the curvature tensor minimising the bending energy (3.1) is $A_y\equiv \overline{A}$.

Taking $\omega =\mathbb {R}^2$, the deformations *y* such that $A_y\equiv \overline{A}$ transform the coordinate lines $x_1 = \hbox {const}$ (parallel to ${\mathsf {e}_2}$) into straight lines, coordinate lines $x_2 = \hbox {const}$ (parallel to ${\mathsf {e}_1}$) into circles of radius

$$\begin{aligned} \frac{1}{\overline{c}}= \frac{2h}{3 \varepsilon } \end{aligned}$$

(covered infinitely many times), and $y(\omega )$ is a cylinder whose cross-section is a circle of radius $1/\overline{c}$ and whose axis is parallel to the image of the coordinate lines $x_1 = \hbox {const}$. The orientation in space of the cylinder is arbitrary, since superposing an arbitrary rigid rotation to *y* leaves $A_y$ unchanged.

**Fig. 1:**
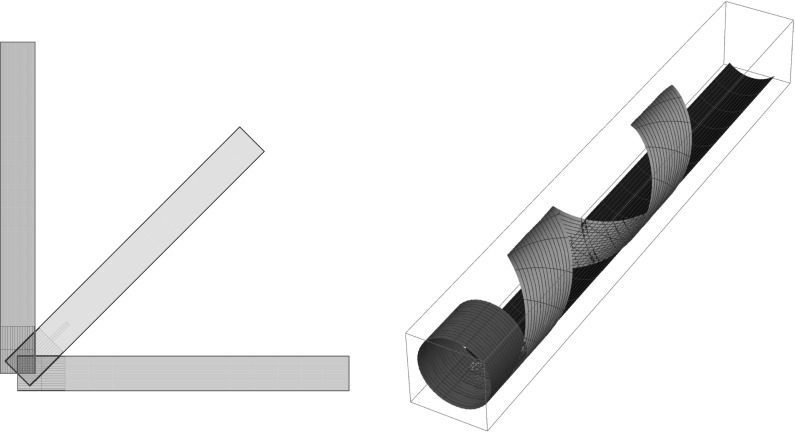
Different shapes obtained with horizontal uniaxal prestretch in the bottom layer, as the angle $\theta$ between the long axis of the ribbon and the pre-stretch axis is varied: $\theta = 0$ (*red*), $\theta = \pi /4$ (*green*), $\theta = \pi /2$ (*blue*). *Left panel*: reference configurations; *right panel*: deformed configurations. (Color figure online)

We now “paint” a subset $\omega _\theta$ of $\omega$ having the shape of a rectangle

$$\begin{aligned} \omega _\theta := R_{\theta } \omega _0 \subset \omega \,, \quad \omega _0=(0,L)\times (-w/2, w/2)\,, \quad 0<w\le L\,, \end{aligned}$$

where $R_{\theta }$ is a rotation of angle $\theta$ and axis ${\mathsf {e}_3}$, and we look for the way a map *y* of minimal bending energy deforms $\omega _\theta$. We obtain in this way different spontaneously curved and twisted ribbon-like shapes $y( \omega _\theta )$, as $\theta$, the angle between the long axis of the rectangle $\omega _\theta$ and the pre-stretch direction ${\mathsf {e}_1}$, is varied. In Fig. 1, we show the cases $\theta =0$, $\theta =\pi /4$, and $\theta =\pi /2$, leading to a coil, a helical ribbon, and a cigar-shaped one reproducing some of the tunable helical ribbons of [8].

We use the terminology “region *painted* on $\omega _\theta$”, rather than “ region *cut* from $\omega _\theta$” to emphasise that, when a physical cut is performed, the stresses along the exposed cut surfaces drop to zero, the pre-stretches in boundary layers near the cut lines are released, and the behaviour of the cut strips resembles that of the painted strips only as long as edge effects are negligible. This will be our working hypothesis here and in what follows. We will study in [7] the regimes of material and geometric parameters in which this assumption leads to acceptable conclusions.

### Equal uniaxial pre-stretches at right angles in the two layers

We have now $\check{E}^*_1=\varepsilon ({\mathsf {e}_2}\otimes {\mathsf {e}_2})$, $\check{E}^*_2= \varepsilon ({\mathsf {e}_1}\otimes {\mathsf {e}_1})$, $\varepsilon >0$. It follows from (3.2) that

4.2 $$\begin{aligned} \overline{A}= \frac{3}{2h} (\check{E}^*_2 - \check{E}^*_1) = \overline{c} ({\mathsf {e}_1}\otimes {\mathsf {e}_1} - {\mathsf {e}_2}\otimes {\mathsf {e}_2})\,, \quad \overline{c}= \frac{3 \varepsilon }{2 h}\,. \end{aligned}$$

The curvature tensor $A_y$ minimising the bending energy (3.1) is obtained as the minimal energy solution of the equilibrium equations (3.7). There are two distinct solutions of equal minimal energy, namely,

4.3 $$\begin{aligned} A_y=A_1=(1-\nu )\overline{c}({\mathsf {e}_1}\otimes {\mathsf {e}_1})\,, \quad \quad A_y=A_2=-(1-\nu )\overline{c}({\mathsf {e}_2}\otimes {\mathsf {e}_2})\,, \end{aligned}$$

describing a pair of cylinders with perpendicular axes, opposite curvature, and whose cross-sections are circles with radius

$$\begin{aligned} \frac{1}{(1-\nu )\overline{c}}= \frac{2h}{3 (1-\nu )\varepsilon }\,. \end{aligned}$$

**Fig. 2:**
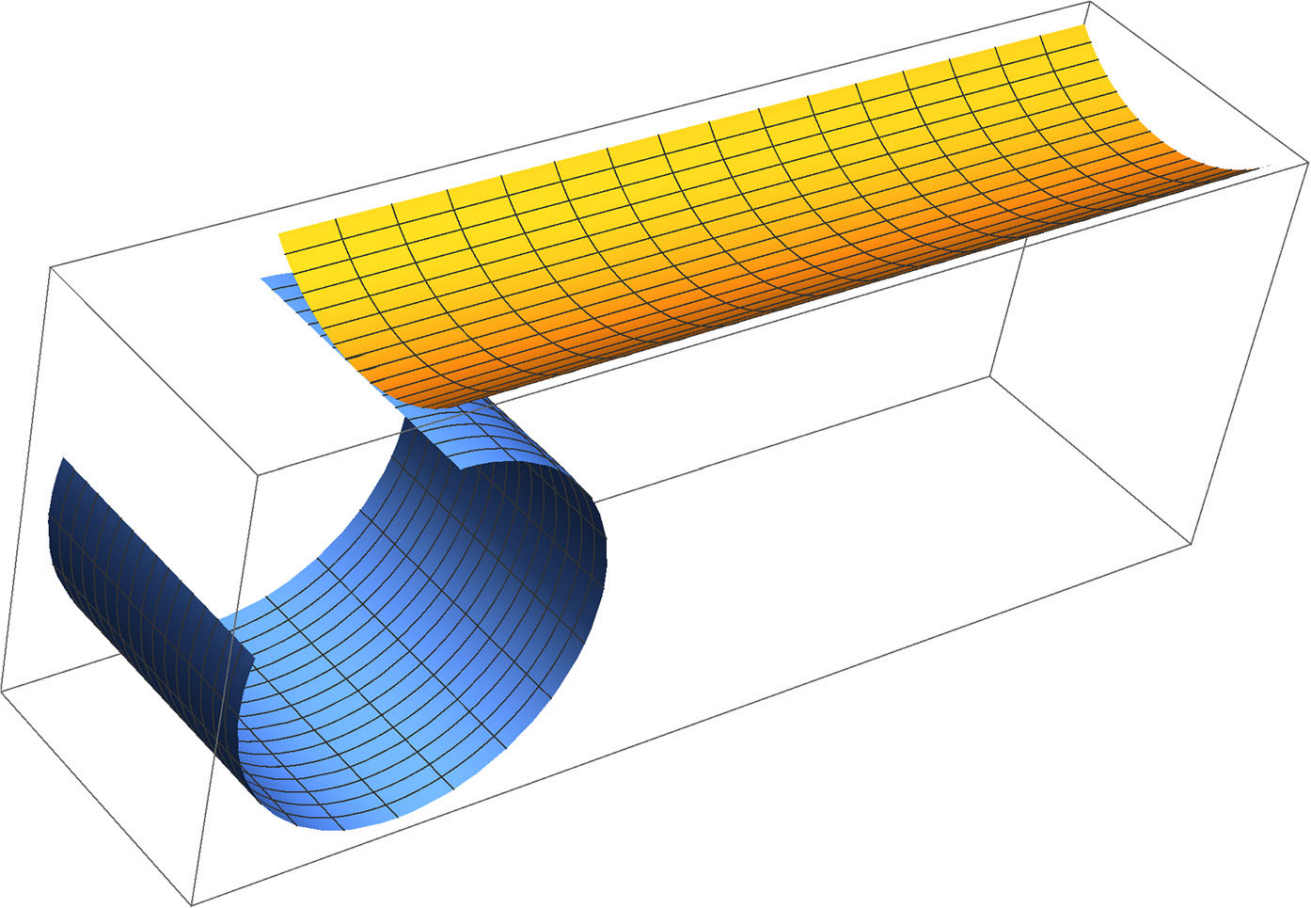
Bistability in the case of equal uniaxial pre-stretch at right angles in the two layers

This means that rectangular strips cut out of $\omega =\mathbb {R}^2$ may show bistability, as in [18]. This behaviour is described pictorially in Fig. 2.

### Equibiaxial pre-stretch in the bottom layer

This is the case $\check{E}^*_1=0$, $\check{E}^*_2= \varepsilon \, \mathrm{I}_2$, $\varepsilon >0$. The target curvature from (3.2) is then

4.4 $$\begin{aligned} \overline{A}= \frac{3}{2h} (\check{E}^*_2 - \check{E}^*_1) = \overline{c} \, \mathrm{I}_2\,, \quad \overline{c}= \frac{3 \varepsilon }{2 h}\,. \end{aligned}$$

In view of the rotational symmetry of the problem, there are infinitely many curvature tensors minimising the bending energy (3.1), namely,

4.5 $$\begin{aligned} A_y=A({\mathsf {e}})=(1+\nu )\,\overline{c}\,({\mathsf {e}}\otimes {\mathsf {e}})\,, \end{aligned}$$

where ${\mathsf {e}}$ is an arbitrary unit vector in the plane of ${\mathsf {e}_1}$ and ${\mathsf {e}_2}$. The mid-plane $\omega$ is mapped to a cylinder whose cross-section is a circle of radius

4.6 $$\begin{aligned} \frac{1}{(1+\nu )\overline{c}}= \frac{2h}{3 (1+\nu )\varepsilon }\,. \end{aligned}$$

and whose generatrix parallel to the cylinder axis may be the image of any straight line in the plane of $\omega$. Put differently, a straight line in the plane of $\omega$ can be mapped by a deformation of minimal bending energy into a helix wrapped around a cylinder of radius (4.6), of arbitrary pitch from zero (in which case it is a circle covered multiple times) to infinity (in which case it is a straight generatrix parallel to the cylinder axis). This means that rectangular strips cut out of $\omega =\mathbb {R}^2$ may behave as shells with zero stiffness to twisting, as in [17]. Of course, in reality the stiffness of the system will not be exactly zero and some preferred configuration will be selected by imperfections and edge effects, see [4, 10, 21]. Nevertheless, the elastic behaviour of these ribbons will be anomalousy soft, when compared to similar sheets with different pre-stretches, or no pre-stretches at all.

**Fig. 3:**
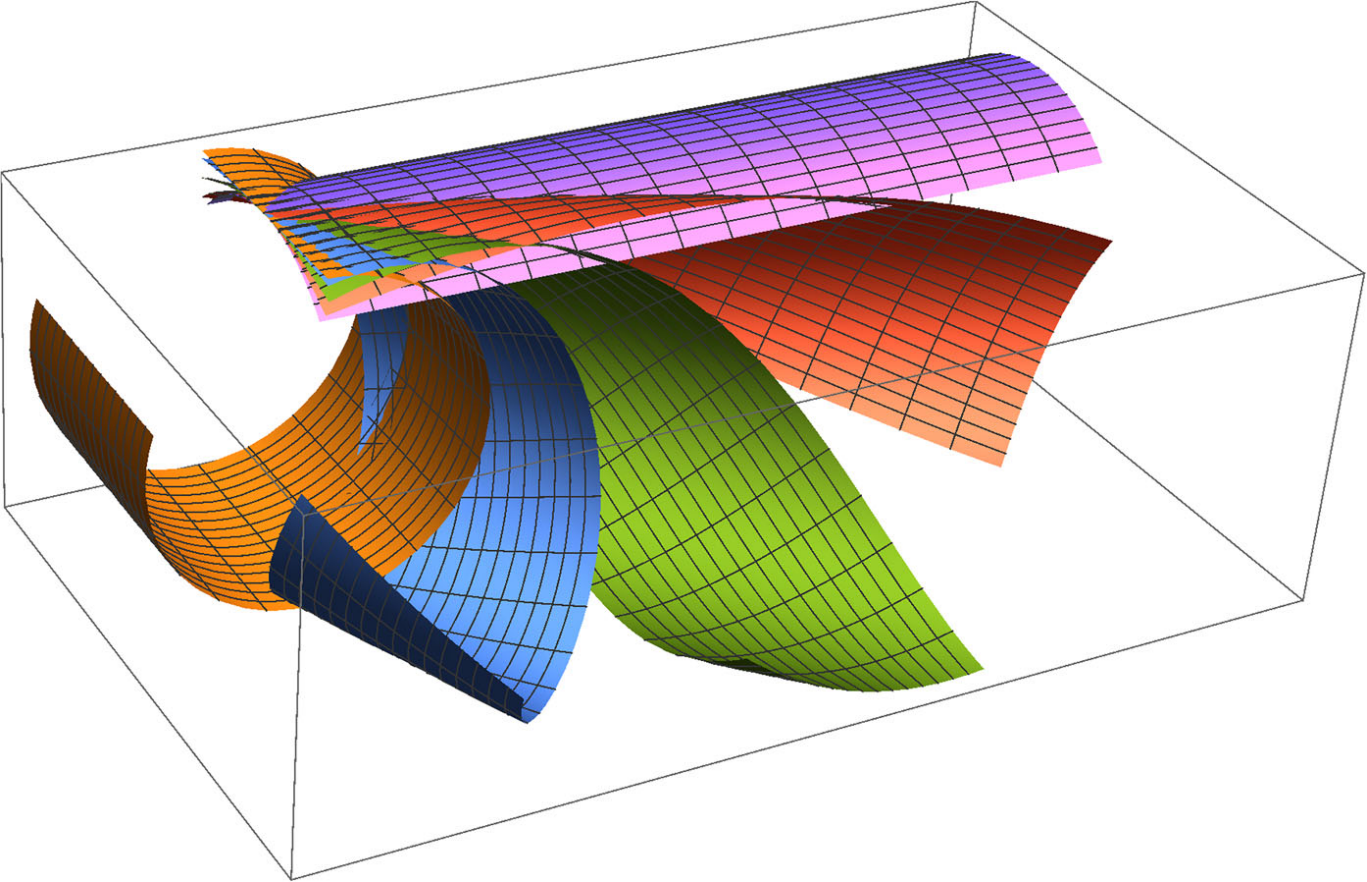
A shell with anomalously low stiffness to twisting emerges in the case of of equibiaxial pre-stretch in only one of the two layers

The soft modes of response of the system discussed above are described pictorially in Fig. 3. Explicit equations describing this continuous family of isometries of $\omega$ of minimal bending energy can be found in [3].

## Discussion

We have shown that pre-stretched latex bilayer sheets can provide us with a very valuable tool to explore shape-programming and surface morphing problems. Based on the predictions of a (nonlinear) bending model, we expect that, by tuning the pre-stretches in the top and bottom halves of the bilayer, we should be able to reproduce a large variety of structural behaviours: from the tunable helical ribbons of [8], to the bistable shells and the shells of zero stiffness of [17, 18].

Several questions and research directions open up as a follow-up of the results of this paper. First, we plan to investigate, both computationally and with laboratory experiments, the reliability of the predictions we have made, which are based on a bending energy functional that arises as a thin film Gamma-limit of a three-dimensional elastic model with spontaneous strains. This will also enable us to go beyond the restrictive scenario of homogeneously curved plates and shells, which can be approached with analytical calculations, and consider the effect of applied loads, imperfections, and edge boundary layers. For some preliminary results in this direction, see [7].

In addition, we plan to export the understanding gained in the study of pre-stretched bilayer sheets, a model system, to more complex physical systems, such as patterned thin films made of hydrogels. This is an interesting system to explore questions regarding the design of not only the equilibrium shapes of thin structures, but also of their time-history (4D printing: where the dimension of time is added to the “standard” three space dimensions [16]). For some preliminary results in this direction, see [1, 24, 25].

## Footnotes

1 We will return to this in [7]
